# The Determinants of Social Connectedness in Europe

**DOI:** 10.1007/978-3-030-60975-7_1

**Published:** 2020-10-07

**Authors:** Michael Bailey, Drew Johnston, Theresa Kuchler, Dominic Russel, Bogdan State, Johannes Stroebel

**Affiliations:** 8 Max Planck Institute for Demographic Research, Rostock, Germany; 9 University of Sheffield, Sheffield, UK; 10 King’s College London, London, UK; 11 Umeå University, Umeå, Sweden; 12 ISTI-CNR, Pisa, Italy; 13 University of Pisa, Pisa, Italy; 14 University of Pisa, Pisa, Italy; 15 Facebook, Menlo Park, USA; 16 Harvard University, Cambridge, USA; 17 New York University, New York, USA

**Keywords:** Social connectedness, Homophily, Border effects

## Abstract

We use de-identified and aggregated data from Facebook to study the structure of social networks across European regions. Social connectedness declines strongly in geographic distance and at country borders. Historical borders and unions—such as the Austro-Hungarian Empire, Czechoslovakia, and East/West Germany—shape present-day social connectedness over and above today’s political boundaries and other controls. All else equal, social connectedness is stronger between regions with residents of similar ages and education levels, as well as between regions that share a language and religion. In contrast, region-pairs with *dissimilar* incomes tend to be more connected, likely due to increased migration from poorer to richer regions.

## Introduction

Social networks shape many aspects of global society including patterns of migration and travel, social mobility, and political preferences. In turn, social networks reflect both past and present political borders and migration patterns, as well as geographic proximity, culture, and other factors. While understanding the determinants and effects of these networks across regions and countries can be informative for a wide range of questions in the social sciences, researchers have traditionally been limited by the scarcity of large-scale representative data on regional social connections.

In this paper, we investigate the spatial structure of social networks in Europe. We measure social networks using de-identified and aggregated data from Facebook, a global online social network.^1^ We construct a measure of social connectedness across European NUTS2 regions—regions with between 800,000 and 3 million inhabitants—which captures the probability that Facebook users located in these regions are Facebook friends with each other. Europe consists of a number of proximate nations, has a relatively high population density, and includes a diversity of areas with distinct cultural and linguistic identities. Each of these factors differentiates Europe from the U.S., which has been the primary focus of prior research on social connectedness. This paper documents the important role that these and other factors play in shaping social connections, and thereby advances our understanding of the determinants of social networks.

We begin by discussing a number of case studies that show the relationship of European social connections with patterns of migration, past and present political borders, geographic distance, language, and other demographic characteristics. We then explore the association between social connectedness and these factors more formally. We find that social connectedness strongly declines in geographic distance: a 10% increase in distance is associated with a 13% decline in social connectedness. Social connectedness also drops off sharply at country borders. Controlling for geographic distance, the probability of friendship between two individuals living in the same country is five to eighteen times as large as the probability for two individuals living in different countries. Furthermore, using a number of 20th century European border changes, we find that this relationship between political borders and connectedness can persist decades after boundaries change. For example, we find higher social connectedness across regions that were originally part of the Austro-Hungarian empire, even after controlling for distance, current country borders, and a number of other relevant factors.

In addition to distance and political borders, we find that regions more similar along demographic measures such as language, religion, education, and age are more socially connected. In particular, social connectedness between two regions with the same most common language is about 4.5 times larger than for two regions without a common language, again controlling for same and border country effects, distance, and other factors. In contrast, we see that pairs of regions with *dissimilar* incomes are more connected. This finding may be explained by patterns of migration from regions with low incomes to regions with high income. This finding in Europe contrasts with prior research that finds a positive relationship between connectedness and income similarity across U.S. counties and New York zip codes [3, 5].

## Data

Our measures of social connectedness across locations builds on de-identified administrative data from Facebook, a global online social networking service. Facebook was created in 2004 and, by the fourth quarter of 2019, had about 2.5 billion monthly active users globally, including 394 million in Europe.

While Facebook users are unlikely to be entirely representative of the populations we study, it has a wide user base. One independent resource estimates 80% of European social media site visits from September 2018 to September 2019 were to Facebook [21]. A separate study found that the number of active accounts on the most used social network in each country, as a share of population, was 66% in Northern Europe, 56% in Southern Europe, 54% in Western Europe, and 45% in Eastern Europe [24]. Another 2018 survey found that the share of adults who used any social networking site in 10 European countries was between 40% and 67% [19].

A related question evolves around the extent to which friendship links on Facebook correspond to real world friendship links. We believe that this is likely. Establishing a Facebook friendship link requires the consent of both individuals, and the total number of friends for a person is limited to 5,000. As a result, networks formed on Facebook more closely resemble real-world social networks than those on other online platforms, such as Twitter, where uni-directional links to non-acquaintances, such as celebrities, are common.

We observed a de-identified snapshot of all active Facebook users from July 2019. We focus on those users who reside in one of 37 European countries and who had interacted with Facebook over the 30 days prior to the date of the snapshot. The 37 countries are the members of the European Union and European Free Trade Association, as well as European Union candidate countries as of 2016; these countries were selected because they have standardized administrative boundaries at the NUTS2 (Nomenclature of Territorial Units for Statistics level 2) level.^2^ NUTS2 regions contain between 800,000 and 3 million people, and are generally based on existing sub-national administrative borders. For example, NUTS2 corresponds to 21 “regions” in Italy, 12 “provinces” in the Netherlands, and a single unit for all of Latvia.

To measure social connections between NUTS2 regions, we follow [3] and construct our measure of $SocialConnectedness_{ij}$ as follows:

1 $$\begin{aligned} SocialConnectedness_{ij} = \frac{FB\_Connections_{ij}}{FB\_Users_i*FB\_Users_j} \end{aligned}$$

Here, $FB\_Connections_{ij}$ is the total number of connections between individuals living in NUTS2 region *i* and individuals living in NUTS2 region *j*. $FB\_Users_i$ and $FB\_Users_j$ are the number of eligible Facebook users in each region. Dividing by the product of regional Facebook users allows us to take into account the fact that we will see more friendship links between regions with more Facebook users. This measure captures the probability that two arbitrary Facebook users across the two countries are friends with each other: if $SocialConnectedness_{ij}$ is twice as large, a Facebook user in region *i* is about twice as likely to be connected with a given Facebook user in region *j*.

We have shown in previous work that this measure of social connectedness is useful for describing real-world social networks. We also documented that it predicts a large number of important economic and social interactions. For example, social connectedness as measured through Facebook friendship links is strongly related to patterns of sub-national and international trade [6], patent citations [3], travel flows [5], investment decisions [13] and the spread of COVID-19 [14]. More generally, we have found that information on individuals’ Facebook friendship links can help understand their product adoption decisions [7] and their housing and mortgage choices [2, 4].

## Determinants of European Social Connectedness

To illustrate the data and explore the factors that shape social connections within Europe, we first highlight the geographic structure of social connections of a few European regions. We provide additional cases studies in the Online Appendix.

**Fig. 1.**
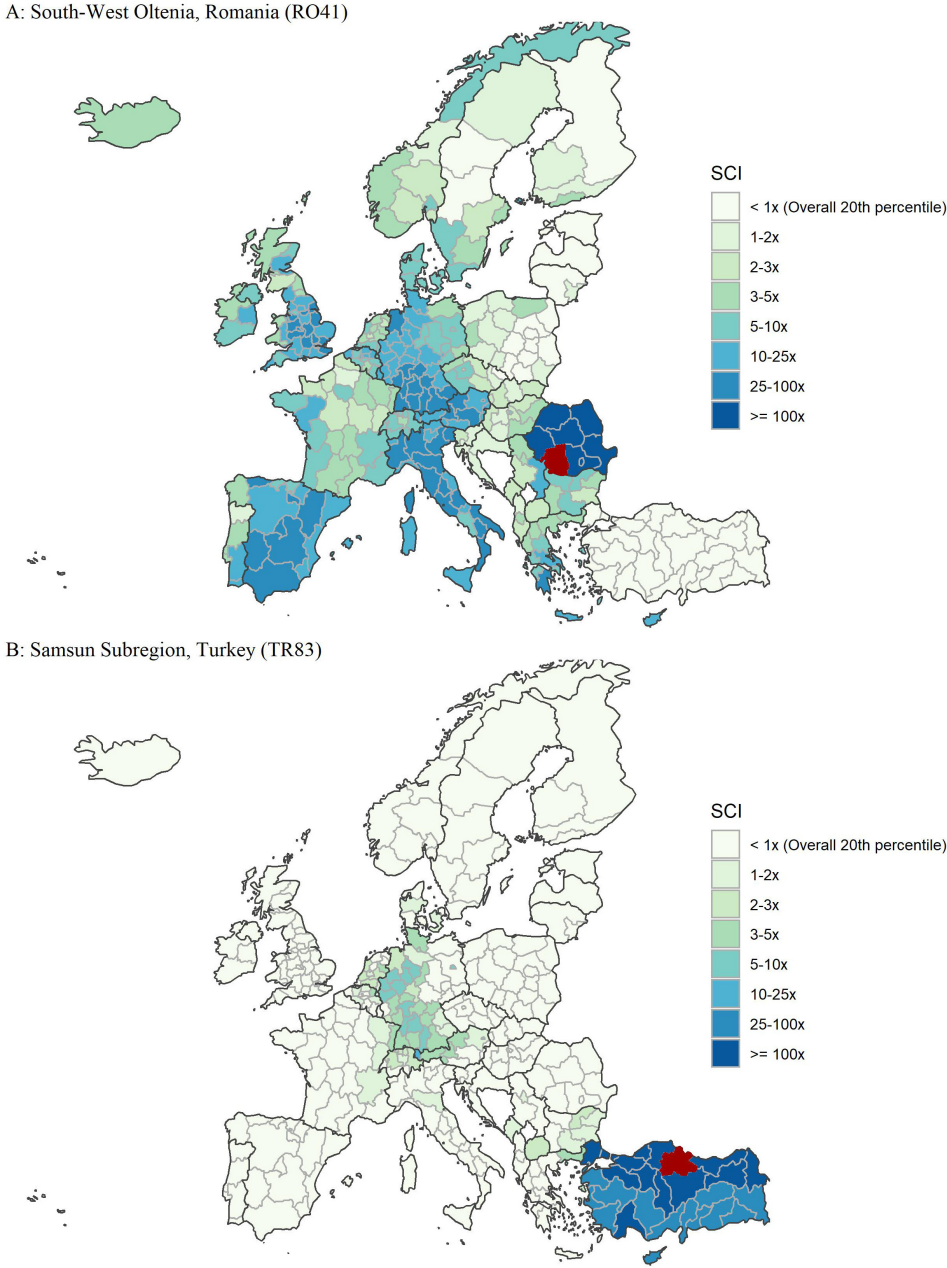
Social Network Distributions in Romania and Turkey (*Note:* Figure shows the relative probability of connection, measured by $SocialConnectedness_{ij}$, of all European regions *j* with two regions *i*: South-West Oltenia, RO (Panel A) and Samsun Subregion, TR (Panel B). The measures are scaled from the 20th percentile of all *i*, *j* pairs in Europe. Darker regions have a higher probability of connection).

Figure 1 maps the social network of South-West Oltenia in Romania in Panel A and the Samsun Subregion in Turkey in Panel B; darker shading indicates greater connectedness. In both examples, the strongest social connections are to nearby regions in the same country. Residents of South-West Oltenia have relatively strong social connections throughout Europe, especially to Italy, Spain, Germany, and the United Kingdom. This is likely related to patterns of migration. Romania became a member of the European Union in 2007, which entitled its citizens to certain freedoms to travel and work in other EU member states. According to a report by the World Bank, between 3 and 5 million Romanians currently live and work abroad, representing around a fifth of the country’s population. The top destination countries in 2017 were Italy, Spain, Germany, the United States, and the United Kingdom [25]. By contrast, Panel B shows that the connections between the Samsun Subregion in Turkey, which is not an EU member state, and other European regions are much weaker. The strongest connections between the Samsun Subregion and other countries are concentrated in western Germany and Berlin, with substantially weaker connections in eastern Germany (former German Democratic Republic). These connections likely reflect the lasting impacts of the West Germany’s 1961–1973 labor recruitment agreement *Anwerbeabkommen* with Turkey, which resulted in many Turkish workers re-settling in West Germany (see the discussion in [1]).

*Assessing Potential Determinants of Social Connectedness.* We next assess the role of the determinants of European social connectedness in a regression framework. To estimate the relationship between various factors and social connectedness between European regions, we estimate the following equation:

2 $$\begin{aligned} \log (SocialConnectedness_{ij}) = \beta _{0} + \beta _1\log (d_{ij}) + X_{ij} + \psi _{i} + \psi _{j} + \epsilon _{ij} \end{aligned}$$

The unit of observation is a pair of NUTS2 regions. The dependent variable is the log of Social Connectedness between regions *i* and *j* (see Eq. 1). The geographic distance is denoted by $\log (d_{ij})$. The log-linear specification follows evidence in [3]. The vector $X_{ij}$ includes measures of similarity and dissimilarity along the following demographic and socioeconomic factors: education (the difference in the share of the population that has only lower secondary education or less), age (the difference in median age), income (the difference in average household income), religion (an indicator for whether the regions have the same most common religion), unemployment (the difference in the average unemployment rate for persons aged 15 to 74 from 2009–2018), language (an indicator for whether the regions have the same language most commonly spoken at home), and industry similarity (the cosine distance between vectors of industry employment shares). In some specifications, we also include indicators that are set equal to one if the two regions are in the same or in bordering countries. All specifications include fixed effects $\psi _{i}$ and $\psi _{j}$ for regions *i* and *j*; this allows us to control for average differences across regions in Facebook usage patterns.

**Table 1.** Determinants of social connectedness across region Pairs

	Dependent variable: log (SocialConnectedness)						
	(1)	(2)	(3)	(4)	(5)	(6)	(7)
log(Distance in KM)	$${-1.318^{***}}$$ (0.046)	$${-0.558^{***}}$$ (0.053)	$${-0.582^{***}}$$ (0.041)	$${-0.572^{***}}$$ (0.038)	$${-0.737^{***}}$$ (0.027)	$${-1.177^{***}}$$ (0.032)	$${-0.591^{***}}$$ (0.031)
Same Country		$${2.896^{***}}$$ (0.077)	$${1.651^{***}}$$ (0.124)				
Border Country			$${0.285^{***}}$$ (0.044)	$${0.340^{***}}$$ (0.046)			
$$\varDelta $$ Share Pop Low Edu (%)			$${-0.013^{***}}$$ (0.002)	$${-0.012^{***}}$$ (0.002)	−0.002 (0.001)	$${-0.007^{**}}$$ (0.003)	−0.000 (0.001)
$$\varDelta $$ Median Age			$${-0.017^{***}}$$ (0.004)	$${-0.021^{***}}$$ (0.004)	0.000 (0.003)	$${-0.014^{***}}$$ (0.005)	0.001 (0.002)
$$\varDelta $$ Avg Income (k €)			$${0.053^{***}}$$ (0.003)	$${0.055^{***}}$$ (0.003)	$${0.015^{***}}$$ (0.002)	$${0.025^{***}}$$ (0.006)	$${0.012^{***}}$$ (0.002)
$$\varDelta $$ Unemployment (%)			−0.000 (0.005)	0.004 (0.005)	$${0.006^{*}}$$ (0.003)	$${0.021^{**}}$$ (0.010)	$${0.007^{*}}$$ (0.004)
Same Religion			0.027 (0.031)	$${0.049^{*}}$$ (0.025)	$${0.044^{***}}$$ (0.013)	$${0.127^{***}}$$ (0.040)	$${0.029^{**}}$$ (0.013)
Same Language			$${1.493^{***}}$$ (0.097)	$${1.548^{***}}$$ (0.120)	$${1.529^{***}}$$ (0.216)	$${2.279^{***}}$$ (0.133)	$${1.909^{***}}$$ (0.107)
Industry Similarity			0.128 (0.169)	0.044 (0.158)	$${0.528^{***}}$$ (0.107)	0.242 (0.199)	$${0.633^{***}}$$ (0.109)
NUTS2 FEs	Y	Y	Y	Y	Y	Y	Y
Indiv. Same Country FEs				Y			
All Country Pair FEs					Y	Y	Y
Sample						Same country	Diff. country
$R^2$	0.490	0.669	0.745	0.775	0.906	0.927	0.839
Number of Observations	75,900	75,900	75,900	75,900	75,900	5,266	70,634

*Note:* Table shows results from Regression 2. The unit of observation is a NUTS2 region pair. The dependent variable in all columns is the log of $SocialConnectedness_{ij}$. Column 1 includes the log of distance and region fixed effects. Column 2 adds a control for regions in the same country. Column 3 incorporates demographic and socioeconomic similarity measures, as well as a control for regions in countries that border. Column 4 adds fixed effect for each same-country pair. Column 5 adds fixed effects for each country pair. Columns 6 and 7 limit the observations to pairs in the same country and pairs in different countries, respectively. Standard errors are double clustered by each region *i* and region *j* in a region pair. Significance levels: *(p<0.10), **(p<0.05), ***(p<0.01).

Table 1 shows regression estimates of Eq. 2. Column 1 includes only the distance measure, $\log (d_{ij})$, and the region fixed effects. A 10% increase in the distance between two regions is associated with a 13.2% decline in the connectedness between those regions. This elasticity is comparable to that observed for U.S. county pairs in [3]. However, the amount of variation in connectedness that distance alone is able to explain is substantially lower in Europe than it is in the United States—in Europe, distance explains 36% of the variation in social connectedness not explained by region fixed effects, while the same number is 65% for the United States. In other words, distance is a less important determinant of social connectedness in Europe than it is in the United States. In column 2, we add the variable indicating whether both regions are in the same country. This “same country effect” explains an additional 18% of the cross-sectional variation in region-to-region social connectedness. The estimated elasticity is larger in magnitude than for same-state indicators in the U.S. county regressions in [3], suggesting that there is a greater drop-off in social connectedness at European national borders than at U.S. state borders.

In column 3, we add differences in demographics and socioeconomic outcomes and an indicator for regions that are in bordering countries as explanatory variables. Regions with the same language and those where residents are more similar in terms of educational attainment and age are more connected to each other. Such “homophily” – more friendship links between similar individuals, regions or countries – has been documented in prior work [2, 3, 5, 15, 16, 22, 23, 27]. Our estimates suggest that social connectedness between two regions with the same language is about 4.5 times larger than for two regions without the same language, even after controlling for same country and border country effects, geographic distance, and other demographic and socioeconomic factors. When we include language and demographic factors, the estimated effect of being in the same country falls (from a coefficient estimate of 2.9 to 1.6) suggesting that some—but not all—of the higher in intra-country connectedness is due to common language and other demographic similarities.

Somewhat surprisingly, we see higher connectedness between regions with larger differences in income, even after controlling for country-pair fixed effects, and both limiting to regions within the same country and limiting to regions in different countries. In some of these specifications, we also see a positive relationship between connectedness and differences in unemployment. These relationships run contrary to findings from prior research that finds positive relationships between connectedness and income similarity across U.S. counties and New York zip codes [3, 5]. A possible explanation is related to the migration patterns suggested by our case studies: migrants are particularly likely to move from regions with low income (or higher unemployment) to regions with higher income (or lower unemployment) and comparatively less likely to move to other low or middle income regions. Hence, we see more migration and more connections between regions with large differences in income versus those with more similar levels of income or unemployment. This finding is particularly interesting in light of a recent and substantial literature on intra-U.S. migration that documents a general decline in moves over the past three decades and the importance of opportunistic moves for the U.S. labor market (for example, [11, 12, 17, 26]). By contrast, much less is known about regional migration flows within Europe, largely due to a lack of comprehensive data. The existing prior research has focused on country-to-country flows [10], the intensity of within country migration [8, 9], or regional net-migration [20]. Our unique data set on connectedness provides insights into region-to-region migration patterns throughout the continent. For example, existing data show that within country moves in Europe are generally less common than in the United States; however, the positive relationship we observe between income dissimilarity and connectedness, compared to the negative relationship observed in the U.S., suggest that there may be higher rates of migration in Europe from less prosperous to more prosperous regions. These are exactly the opportunistic moves that increase labor market dynamism.

Column 4 adds fixed effects for each same-country pair, and column 5 adds fixed effects for every country pair. The magnitude of the coefficient on income dissimilarity falls, consistent with country-level migration flows explaining some of the connectedness between regions with dissimilar incomes; however, even holding average connectedness across country pairs fixed, social connectedness is stronger between regions with more different incomes. Columns 6 and 7 limit to pairs of regions in the same and in different countries, respectively. Social connectedness declines in geographic distance more within countries than across countries: a 10% greater geographic distance between regions within the same country implies a 11.7% decrease in social connectedness, whereas a 10% greater geographic distance between regions in different countries implies only a 5.9% decrease in connectedness (conditional on the other controls).

**Fig. 2.**
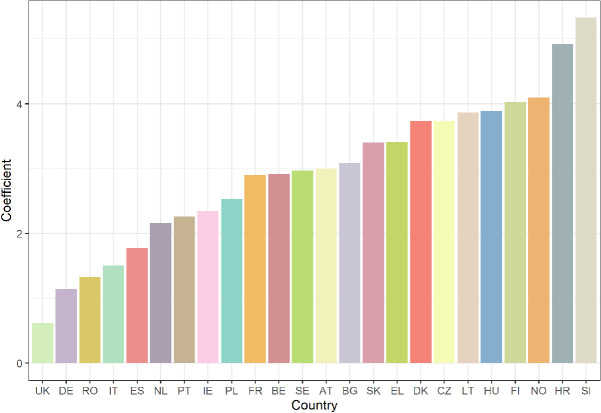
Connectedness within European Countries (*Note:* Figure shows coefficients of the individual same-country effects from the regression reported in column 4 of Table 1. The coefficients are roughly the additional connectedness that is associated with two regions being part of the same country, for each country. Higher values are indicative of stronger within-country connectedness (after controlling for certain demographic and socioeconomic effects). The labels on the x-axis are the two-letter prefix of each country’s NUTS codes).

*Strength of Within-Country Connectedness.* So far, we have shown that, on average, regions in the same country are more connected than regions in different countries that are similarly far apart. We next explore the extent of heterogeneity in this within-country effect on connectedness. We do so by comparing the coefficients on the individual same-country effects estimated in column 4 of Table 1, which capture the additional connectedness associated with two regions being part of the same country. Figure 2 shows these coefficients plotted for all countries with two or more NUTS2 regions. Higher values are indicative of stronger within-country social connectedness. Within-country connectedness is generally stronger for countries with smaller populations, such as Slovenia and Croatia, than for countries with larger populations, such as the United Kingdom and Germany. There are also noticeable differences between countries of similar sizes. For example, the United Kingdom and France have roughly equal populations, yet two regions in France are on average 18 times more connected than two similarly situated regions in Europe, whereas two regions in the United Kingdom are only 1.8 times more connected. There are several possible reasons for such differences, such as historical patterns (e.g., did the nations unite at different times?), geography (e.g., are there physical barriers that separate parts of the nations?), or modern government structures (e.g., do sub-regional governments have greater autonomy in some countries than in others?). Determining the relative importance of these factors is an exciting avenue for future research.

*Relationship Between Historical Borders and Connectedness.* Next, we take a more detailed look at the relationship between historical political boundaries and today’s social connectedness. Information on national borders in 1900, 1930, 1960, and 1990 comes from [18].^3^ Table 2 adds additional variables based on these historical borders to the analysis in Table 1. Column 1 uses all of the same controls as column 4 in Table 1 except log(Distance); throughout this table, we instead use 100 dummy variables representing percentiles of the distribution of distance to avoid picking up non-linearities in the relationship between geographic distance and historical borders.^4^ Columns 2 to 5 add indicators based on national borders at the start of 1990, 1960, 1930, and 1900, respectively.

We look at several major European border changes dating back to the early 20th century, showing that present-day connectedness is higher between regions that have been part of the same country in the past. This result is *in addition* to the effects of being in the same country today, being in bordering countries today, region-to-region distance, and all the demographic and socioeconomic controls in Table 1. The largest increases in connectedness from having been part of the same country are associated with the most recent border changes. For example, two regions in former Czechoslovakia (which split in 1993) are more than 19 times more connected on average than similar region pairs in other countries. Likewise, two regions in former Yugoslavia (which split in the early 1990s) are more than 13 times more connected. Patterns of social connectedness are also related to country borders prior to the 1990–1991 fall of the Soviet Union. Specifically, connectedness between regions that were both within East Germany is more than 2 times higher than connectedness between other similar region pairs in Germany. Pairs of regions in the three countries in our data that were former republics of the Soviet Union—Estonia, Latvia, and Lithuania—are also 6 times more connected than similar region pairs.

**Table 2.** Historical determinants of social connectedness

	Dependent variable: log(SocialConnectedness)				
	(1)	(2)	(3)	(4)	(5)
		1990	1960	1930	1900
Border Country	$$0.418^{***}$$ (0.045)	$$0.399^{***}$$ (0.045)	$$0.392^{***}$$ (0.045)	$$0.372^{***}$$ (0.045)	$$0.310^{***}$$ (0.043)
Both Czechoslovakia		$$3.525^{***}$$ (0.217)	$$3.529^{***}$$ (0.217)	$$3.541^{***}$$ (0.216)	$$ 2.945^{***}$$ (0.217)
Both Yugoslavia		$$3.108^{***}$$ (0.105)	$$3.110^{***}$$ (0.105)	$$3.123^{***}$$ (0.105)	$$2.616^{***}$$ (0.114)
Both West Germany		0.006 (0.046)	0.005 (0.046)	0.015 (0.044)	−0.005 (0.043)
Both East Germany		$$1.088^{***}$$ (0.053)	$$1.092^{***}$$ (0.053)	$$1.072^{***}$$ (0.055)	$$1.124^{***}$$ (0.050)
Both Soviet Union		$$1.884^{***}$$ (0.080)	$$1.874^{***}$$ (0.081)	$$1.882^{***}$$ (0.081)	$$2.052^{***}$$ (0.077)
Both United Kingdom 1960			$$1.015^{***}$$ (0.155)	$$1.016^{***}$$ (0.156)	$$0.998^{***}$$ (0.157)
Both Germany 1930				$$0.465^{***}$$ (0.104)	$$0.159^{**}$$ (0.063)
Both Austro-Hungarian Empire 1900					$$0.920^{***}$$ (0.111)
Both German Empire 1900					$$0.492^{***}$$ (0.074)
Both United Sweden-Norway					$$2.057^{***}$$ (0.123)
Distance Controls	Y	Y	Y	Y	Y
Table 1 Controls	Y	Y	Y	Y	Y
NUTS2 FEs	Y	Y	Y	Y	Y
Indiv. Same Country FEs	Y	Y	Y	Y	Y
$R^2$	0.784	0.790	0.791	0.792	0.801
Number of Observations	75,900	75,900	75,900	75,900	75,900

*Note:* Table shows results from Regression 2 with added historical country borders controls $X_{ij}$. The unit of observation is a NUTS2 region pair. The dependent variable in all columns is the log of $SocialConnectedness_{ij}$. Every column includes controls for same country, region *i*, and region *j* effects. Column 1 is the same as column 4 of Table 1, except with 100 dummy variables representing percentiles of distance instead of log(distance). Columns 2, 3, 4, and 5 add controls for certain historical borders in 1990, 1960, 1930, and 1900, respectively. Coefficients for the demographic and socioeconomic controls in Table 1 are excluded for brevity. Standard errors are double clustered by each region *i* and region *j* in a region-pair. Significance levels: *(p<0.10), **(p<0.05), ***(p<0.01).

Borders dating back to earlier in the 20th century appear to have weaker, though still economically and statistically significant relationships with present-day social connectedness. In the early 20th century, the United Kingdom controlled both Malta and Cyprus (the two became independent in 1960 and 1964, respectively). A pair of regions in Malta, Cyprus, or the UK are twice as connected as a similarly situated regional pair, again, over and above modern country borders. The borders of Germany in 1930 were also different than today: the country included the Liege region in modern Belgium and a number of regions in modern Poland; on the other hand, it did not include the Saarland—a formerly independent nation within modern Germany. We find a 46% increase in connectedness between regions that were part of 1930 Germany (but are not part of the same country today).

Finally, we look at three national borders that changed before or shortly after the first World War: the Austro-Hungarian Empire, the German Empire, and the United Kingdoms of Sweden and Norway. In 1900, the Austro-Hungarian Empire stretched across much of central and eastern Europe, encompassing part or all of modern Austria, Hungary, Czech Republic, Slovakia, Slovenia, Croatia, Romania, Poland, and Italy. After adding our present-day controls, we find that two regions within this empire are more than 90% more connected than a pair of otherwise similar regions. Compared to modern Germany, the German Empire in 1900 controlled large parts of modern Poland (even more so than 1930 Germany) and the Alsace region of France. We find that having been part of the German Empire in 1900 is associated with a 50% increase in present-day social connectedness, again controlling for both the effects of the modern German borders and 1930 German borders. It is interesting that the regression primarily loads on the older 1900 borders, while the coefficient for the 1930 borders decreases. One possible explanation is the period of time the borders were in effect: whereas the 1930 German borders were effective only in the 20 year interwar period (and indeed changed even during that period), the 1900 borders essentially remained unchanged for nearly 50 years between 1871 to 1918. Lastly, from 1814 to 1905 the lands of present-day Sweden and Norway were united under a common monarch as the United Kingdoms of Sweden and Norway. A pair of regions within this union are more than 7 times more connected today than similarly situated regions in otherwise similar country-pairs. As with all of our analyses, the historical patterns we observe are correlations rather than necessarily causal and may also capture the effect of other factors that relate to historical borders that we do not explicitly control for.

## Conclusion

We use de-identified and aggregated data from Facebook to better understand social connections in Europe. We find that social connectedness declines substantially in geographic distance and at country borders. Using a number of 20th century border changes (such as the breakups of the Austro-Hungarian Empire and Czechoslovakia), we find that the relationship between political borders and social connectedness can persist decades after boundaries change. We also find evidence of homophily in Europe, as connections are stronger between regions with residents of similar ages and education levels, as well as between those that share a language and religion. However, region pairs with *dissimilar* incomes are more connected, likely due to migration from poorer to richer regions.

In our Online Appendix, we explore a number of *effects* of social connections across countries. We first look at the relationship between social connectedness and travel flows. We find that a 10% increase in social connectedness between two regions is associated with a 12% to 17% increase in the number of passengers that travel between the regions by train. This result persists even after controlling for geographic distance and travel time, by train and car, between the central points of the regions. We highlight that this result provides empirical support for a number of theoretical models suggesting social networks play an important role in individuals’ travel decisions. It also provides strong evidence that the patterns of social connectedness correspond to real-world social connections.

In the Online Appendix, we also study how variation in the degree of connectedness of European regions to other countries is reflected in political outcomes. We first document substantial variation across European regions in the share of friendship links that are to individuals living in other European countries: at the 10th percentile of the distribution, less than 4.1% of connections are to individuals in a different country, compared to over 19.7% at the 90th percentile. We then explore the relationship between this variation and the share of a region’s residents that hold Eurosceptic beliefs or that vote for Eurosceptic political parties. According to both measures, we find that Euroscepticism decreases with the share of a region’s connections that to regions in a different European country. Specifically, a 1% point increase in the share of a region’s connections that are to individuals outside of their home country is associated with a 0.5% point increase in the share of residents who trust the E.U. and a 0.76% point decrease in the share that voted for an anti-E.U. political party. These results persist, but become weaker (0.25 and −0.54% points, respectively), after adding controls for the share of residents living in the region who are born in other European countries as well as the regional average income and unemployment rate, and the shares of employment in manufacturing, construction, and professional sectors. While causality behind this result is hard to establish, it is consistent with the theory that exposure to other European countries increases pro-European views, a narrative that lies behind the creation of programs such as the Erasmus European student exchange.

**Our Online Appendix & Additional Results are available at:** http://arxiv.org/abs/2007.12177

## Footnotes

1 The European social connectedness data that we compile and use in this project is accessible at http://data.humdata.org/dataset/social-connectedness-index. See [3] for information on county-level U.S. social network data and [5] for zip code-level data in the New York Combined Statistical area. Additional results using the European data are available in our Online Appendix, located at: http://arxiv.org/abs/2007.12177.

2 Specifically the list of countries is: Albania, Austria, Belgium, Bulgaria, Croatia, Cyprus, Czech Republic, Denmark, Estonia, Germany, Greece, Finland, France, Hungary, Iceland, Ireland, Italy, Latvia, Lichtenstein, Lithuania, Luxembourg, Malta, Montenegro, the Netherlands, North Macedonia, Norway, Poland, Portugal, Romania, Serbia, Spain, Slovakia, Slovenia, Sweden, Switzerland, Turkey, and the United Kingdom.

3 In cases when a modern NUTS2 region spans two historical countries, we classify the region as part of the country for which it had a greater land area overlap.

4 In general, the historical coefficients in Table 2 do not change, or become slightly larger, when using log(Distance).
